# The mitochondrial respiration signature of the bovine blastocyst reflects both environmental conditions of development as well as embryo quality

**DOI:** 10.1038/s41598-023-45691-2

**Published:** 2023-11-08

**Authors:** Jessica Kurzella, Dennis Miskel, Franca Rings, Ernst Tholen, Dawit Tesfaye, Karl Schellander, Dessie Salilew-Wondim, Eva Held-Hoelker, Christine Große-Brinkhaus, Michael Hoelker

**Affiliations:** 1 Institute of Animal Sciences, Animal Breeding, University of Bonn, Endenicher Allee 15, 53115 Bonn, Germany; 2 Department of Biomedical Sciences, Animal Reproduction and Biotechnology Laboratory, Colorado State University, 3105 Rampart Rd, Fort Collins, CO 80521 USA; 3 Department of Animal Science, Biotechnology and Reproduction of Farm Animals, Georg-August-University Goettingen, Burckhardtweg 2, 37077 Göttingen, Germany

**Keywords:** Biotechnology, Developmental biology

## Abstract

The major limitation of the widespread use of IVP derived embryos is their consistent deficiencies in vitality when compared with their ex vivo derived counterparts. Although embryo metabolism is considered a useful metric of embryo quality, research connecting mitochondrial function with the developmental capacity of embryos is still lacking. Therefore, the aim of the present study was to analyse bovine embryo respiration signatures in relation to developmental capacity. This was achieved by taking advantage of two generally accepted metrics for developmental capacity: (I) environmental conditions during development (vivo vs. vitro) and (II) developmental kinetics (day 7 vs. day 8 blastocysts). Our study showed that the developmental environment affected total embryo oxygen consumption while different morphokinetics illustrating the embryo qualities correlate with maximal mitochondrial respiration, mitochondrial spare capacity, ATP-linked respiration as well as efficiency of ATP generation. This respiration fingerprint for high embryo quality is reflected by relatively lower lipid contents and relatively higher ROS contents. In summary, the results of the present study extend the existing knowledge on the relationship between bovine embryo quality and the signature of mitochondrial respiration by considering contrasting developmental environments as well as different embryo morphokinetics.

## Introduction

In recent years, the production and transfer of in vitro produced (IVP)embryos gained importance for the selection of genetically superior cattle and faster breeding success to support sustainable agriculture^1^. One major limitation of the widespread use of IVP bovine embryos, however, is their deficiencies in terms of vitality compared with their ex vivo derived counterparts^2^. More specifically, during the last decades, it has been demonstrated that IVP embryos differ morphologically, metabolically as well as on the molecular level from their in vivo produced counterparts, resulting in a lower viability. There is consensus that the development rate to the blastocyst stage is highly determined by the oocyte whereas the quality of the developed blastocysts depends on the surrounding environment, i.e. culture conditions^3^. Unfortunately, no major improvements of culture conditions have been realised and thus, the success rates for the IVP of bovine embryos have not improved markedly over the last two decades. Embryo development rates remained on a constant level with blastocyst rates, rarely exceeding 40%, as reviewed by Lonergan and Fair^4^. Likewise, development to term after transfer of IVP bovine embryos did not increase within the last two decades, with rates ranging between 35 and 55%being significantly lower compared to pregnancy rates after the transfer of in vivo derived embryos, as reviewed recently by Hansen^5^. But not only blastocyst source (Vivo vs. Vitro), but also developmental kinetics of IVP derived embryos, correlates with pregnancy rates after transfer to recipients^6^. Bovine embryos cleaving earlier developed to the blastocyst stage at higher rates compared to those cleaving later^7^. Accordingly, after freeze-thawing, fast developing IVP derived embryos reaching blastocyst stage on day 7 post fertilization result in significantly higher pregnancy rates after transfer to recipients compared to those reaching blastocyst stage no earlier than day 8^8^. Although generally accepted, the mechanism behind this is not well understood. Taken together, the increasing importance of the IVP of bovine embryos in commercial cattle breeding programs demands improvement with respect to in vitro culture conditions to improve the viability of embryos derived from IVP. However, the mode by which contrasting environments of development and embryo morphokinetics determine or affect embryo quality or viability is barely studied.

Research of genetic factors shows a link between development and metabolic factors. In a previous study, bovine 2-cell stage embryos that developed to the blastocyst stage displayed a typical gene expression fingerprint relating to pathways involving energy metabolism and oxidative phosphorylation^9^. These results were further confirmed by follow-up studies demonstrating typical expression trends of genes related to energy metabolism and oxidative phosphorylation both in individual bovine IVP embryos^10^ and in ex vivo derived embryos^11,12^. Importantly, genes related to energy metabolism and oxidative phosphorylation were not only differentially expressed between competent and none-competent embryos but also between ex vivo and in vitro derived embryos^13^. Therefore, embryo respiration might be the key to explain contrasting viabilities both for in vivo vs. in vitro derived bovine embryos as well as between early and late developing blastocysts.

Bearing in mind that oxidative phosphorylation takes place within mitochondria, it is not surprising that mitochondria became a recent focus of interest. Ge and colleagues, showed a relationship between mitochondrial characteristics and reproductive outcome^14^. In agreement, studies demonstrated that differential regulation of mitochondrial genes between in vitro and in vivo derived blastocysts^15^. and mitochondrial function was key in limiting embryonic development^16^. Studies enlightening interconnections between mitochondrial function and developmental capacity of oocytes and embryos are still lacking though. In that regard, several parameters of metabolism were suggested as usable biomarkers for prediction of embryonic quality, morphokinetics and developmental capacity^17^. However, studies dealing with direct metabolic comparisons between in vivo and in vitro derived embryos to establish a deeper understanding in terms of interdependencies between mitochondrial features and embryo viability are rare. Therefore, a closer look at mitochondrial respiration signatures might further explain contrasting viabilities of bovine embryos. Even earlier, several specific mitochondrial features, including mitochondrial respiration, mitochondrial spare capacity (maximal mitochondrial respiration related to mitochondrial respiration) and efficiency of ATP generation (ATP-linked respiration related to mitochondrial respiration) were proposed for gaining a deeper understanding of mitochondrial function during embryo development in mice, rabbits^18,19^ and the bovine^20,21^. Some of these mitochondrial parameters were recently shown to be determinable via an extracellular FLUX analyser^22^. However, no study on bovine embryos has been conducted taking advantage of this useful technical option of analysing oxygen consumption signatures of bovine embryos representing contrasting developmental capacities so far.

Therefore, the aim of the present study was to analyse bovine embryo respiration signatures in relation to developmental capacity. This was achieved by taking advantage of two generally accepted metrics for developmental capacity: (I) environmental conditions during development (vivo vs. vitro) as well as (II) developmental kinetics (day 7 vs. day 8 blastocysts).

## Results

**Figure 1:**
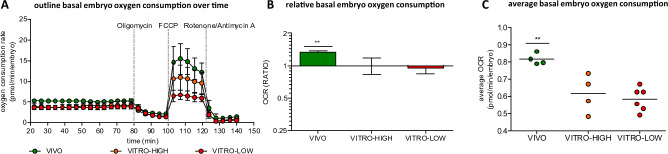
Total embryo oxygen consumption rate (OCR) of contrasting groups of expanded blastocysts (VIVO vs. VITRO-HIGH vs. VITRO-LOW) over time during stress test (**A**). Ratio of total embryo oxygen consumption (Mean ± SD, **B**) as well as total average oxygen consumption of one embryo (**C**) illustrates significant differences (p < 0.05) between embryos of contrasting groups.

In total, 32 in vivo derived expanded blastocysts (EX) serving as the gold standard (VIVO) were allocated to pools of 8 embryos (n = 4) for metabolic measurement. Contrastingly, 32 IVP derived embryos reaching expansion by day 7 (VITRO-HIGH), serving as the model for an alternative developmental environment, were also allocated to pools of 8 embryos (n = 4). As a model for low embryo quality (VITRO-LOW), 48 IVP derived embryos reaching blastocyst expansion after 8 days of culture (6 replicates, each 8 embryos). The entire outline for the measurement of oxygen consumption over time followed by metabolic testing via serial injections of Oligomycin, FCCP (Carbonyl cyanide 4-(trifluoromethoxy) phenylhydrazone) and Rotenone Antimycin A are illustrated in Fig. 1A. All raw data of this study are included in the supplementary information files (Supplementary Data 1).

### Developmental environment affects total embryo oxygen consumption

The results of our analysis showed that developmental environment significantly (p < 0.05) affects total embryo oxygen consumption. Expanded blastocysts of group VIVO displayed 1.3-fold higher oxygen consumption rates (OCR) compared with both VITRO-HIGH and VITRO-LOW groups. In contrast, there was no difference between expanded IVP blastocysts of high and low quality (Fig. 1B). When calculating the absolute OCR values of the embryo group on an average OCR value per embryo per well, average OCR per embryo of group VIVO consumed 0.81 ± 0.032 pmol O_2_ per minute, whereas embryos of the VITRO-HIGH and VITRO-LOW groups consumed significantly lower amounts (0.61 ± 0.110 and 0.58 ± 0.068 pmol O_2_ per minute and embryo, respectively) (Fig. 1C).

### Developmental environment and embryonic quality do not correlate with mitochondrial respiration

**Figure 2:**
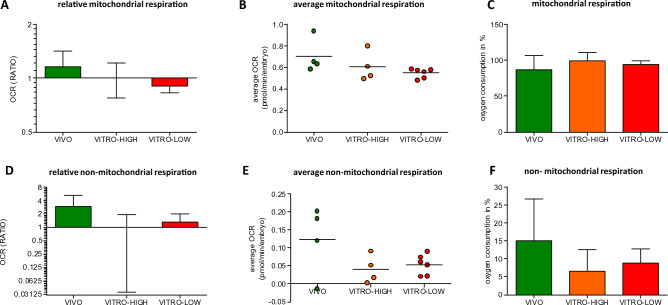
Ratio of mitochondrial respiration (Mean ± SD, **A**) as well as average mitochondrial respiration of one embryo (**B**) illustrates no difference between embryos of contrasting groups. Likewise, proportion of mitochondrial respiration relative to total embryo oxygen consumption (Mean ± SD) did not differ among groups (**C**). Ratio of Non-mitochondrial respiration (Mean ± SD, **D**) as well as average non-mitochondrial respiration of one embryo (**E**) and proportion of mitochondrial respiration (Mean ± SD) relative to total embryo oxygen consumption (**F**) did not differ among groups.

When dissecting total embryo oxygen consumption into mitochondrial and non-mitochondrial respiration via the mitochondrial stress test, the relative values for mitochondrial respiration of VIVO embryos tended to be higher compared to those of the VITRO-HIGH group (116.0% vs. 100.0%). Likewise, a higher embryo quality (VITRO-HIGH vs. VITRO-LOW) went along with slightly higher relative values for mitochondrial respiration (1.0 vs. 0.90) as presented in Fig. 2A (as RATIO). Accordingly, the average mitochondrial respiration per embryo of the VIVO group was 0.707 ± 0.140 pmol per minute, higher than that of embryo of VITRO-HIGH and VITRO-LOW groups (0.609 ± 0.118 and 0.549 ± 0.041 pmol) as shown in Fig. 2B. The mitochondrial proportion of total embryo oxygen consumption was similar for VIVO, VITRO-HIGH and VITRO-LOW, reaching 86.7–99.1% (Fig. 2C). Absolute values for non-mitochondrial respiration (as RATIO) tended to be lower in VITRO-HIGH and VITRO-LOW groups compared to the VIVO group. Accordingly, the average non-mitochondrial respiration per embryo of the VIVO group was 0.123 ± 0.039 pmol per minute, higher than that of embryos of VITRO-HIGH and VITRO-LOW groups (0.041 ± 0.039 and 0.054 ± 0.024 pmol/embryo/minute) as shown in Fig. 2E. Finally, the non-mitochondrial proportion of total embryo oxygen consumption was similar for VIVO, VITRO-HIGH and VITRO-LOW embryos (6.5–15.0%) (Fig. 2F).

### Development environment and embryo quality influences the mitochondrial oxidative potential

**Figure 3:**
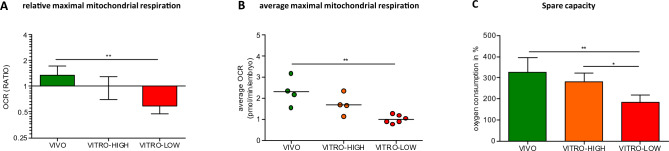
Ratio of maximal mitochondrial respiration (Mean ± SD, **A**) as well as average maximal mitochondrial respiration of one embryo (**B**) illustrates significant (p < 0.05) differences between embryos of contrasting groups. Likewise, the proportion of maximal mitochondrial respiration relative to mitochondrial respiration, termed as mitochondrial spare capacity (Mean ± SD) differed significantly among groups (**C**).

The mitochondria of VIVO embryos consumed 1.7-fold (p < 0.01) more maximal oxygen compared with the mitochondria of VITRO-LOW embryos. Likewise, the mitochondria of the VITRO-HIGH group consumed 1.2-fold more maximal oxygen compared with those of VITRO-LOW embryos (Fig. 3A). The average maximal respiration per embryo of the VIVO and VITRO-HIGH groups reached maximal mitochondrial respiration rates of 2.30 ± 0.628 and 1.70 ± 0.499 pmol per embryo and minute, respectively, but this difference was not statistically significant. Conversely, mitochondria of VIVO embryos reached a significantly higher (p < 0.01) average maximal oxygen consumption per embryo compared to mitochondria of VITRO-LOW embryos (2.30 ± 0.628 vs. 1.007 ± 0.195 pmol per embryo and minute, respectively) as indicated in Fig. 3B. Further examination of mitochondrial respiration showed that mitochondrial spare capacity was not significantly different between VIVO and VITRO-HIGH groups (333.6% vs. 279.0%), whereas the VIVO group displayed a significantly higher mitochondrial spare capacity when compared with the VITRO-LOW group (333.6% vs. 184.0%, respectively). Moreover, there was a significant difference between embryos of VITRO-HIGH vs. VITRO-LOW (Fig. 3C).

### Embryo quality correlates with oxygen utilization for ATP-linked respiration and efficiency of ATP generation

**Figure 4:**
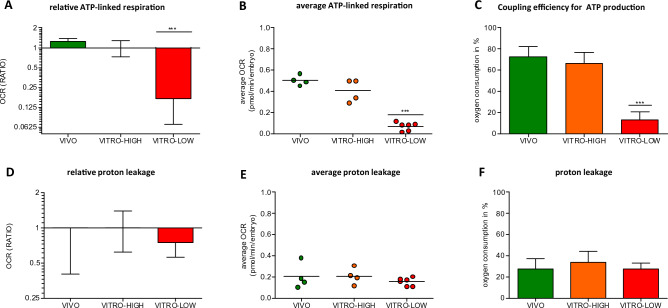
Ratio of mitochondrial respiration utilized for ATP generation (ATP-linked respiration) (Mean ± SD, (**A**) as well as average ATP-linked respiration of one embryo (**B**) illustrates significant (p < 0.05) differences between embryos of contrasting groups. Likewise, proportion of ATP-linked respiration, termed efficiency of ATP generation (Mean ± SD) differed significantly among groups (**C**). Ratio of mitochondrial respiration lost by proton leakage (Mean ± SD, **D**) as well as average mitochondrial respiration lost by proton leakage of one embryo (**E**) and proportion of mitochondrial respiration lost by proton leakage (Mean ± SD) relative to mitochondrial respiration, termed relative proton leak, (**F**) did not differ among groups.

Higher embryo quality (VITRO-LOW vs. VITRO-HIGH) went along with an approximately 1.3-fold higher oxygen consumption for ATP-linked respiration (Fig. 4A). In detail, VITRO-HIGH embryos utilized significantly (p < 0.0001) higher average amounts of oxygen for ATP linked respiration per embryo compared to the VITRO-LOW embryos (0.404 ± 0.109 vs. 0.068 ± 0.039 pmolper embryo and minute). In contrast, the average ATP-linked respiration per embryo of the VITRO-HIGH group did not differ from those of the VIVO group (0.404 ± 0.109 vs. 0.503 ± 0.048 pmol/embryo/minute) as shown in Fig. 4A and B. Accordingly, the efficiency of ATP generation, defined as a proportion of ATP-linked respiration relative to basal mitochondrial respiration, did not differ between embryos of the VIVO and VITRO-HIGH groups (72.7% vs. 66.1%) as illustrated in Fig. 4C. Embryos of the VITRO-HIGH group, however, demonstrated a significantly higher coupling efficiency compared to embryos of the VITRO-LOW group (66.1%vs.12.7%), as shown in Fig. 4C. Finally, when considering the efficiency of the metabolic activity of cells, there was no significant difference between the VIVO, VITRO-HIGH and VITRO-LOW groups in terms of the relative values for proton leakage (Fig. 4D). Likewise, the average oxygen consumed due to proton leakage per embryo (0.204 ± 0.120 vs. 0.206 ± 0.078 vs. 0.154 ± 0.038 pmol per embryo and minute) and relative proton leakage to mitochondrial respiration did not differ between groups, as demonstrated in Fig. 4E,F.

### The content of intracellular lipids and reactive oxygen species reflect embryo quality

**Figure 5:**
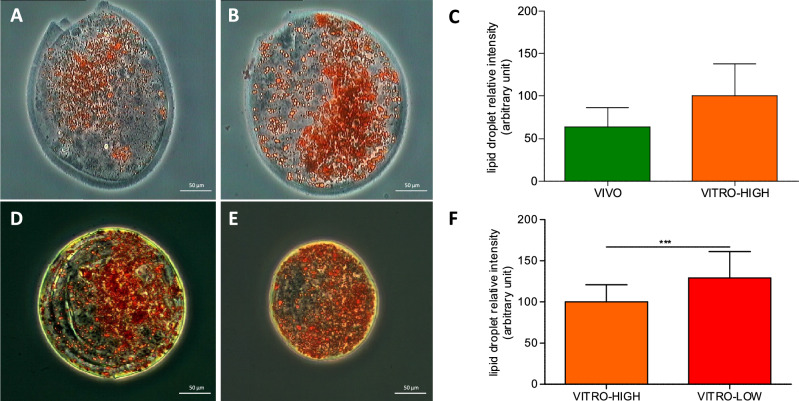
Representative pictures of embryos of VIVO (**A**) and VITRO-HIGH (**B**) groups stained for quantification of lipid droplets (Oil-Red). Ratio of lipid droplets (Mean ± SD) did not show any difference (**C**). Representative pictures of embryos of VITRO-HIGH (**D**) and VITRO-LOW (**E**) groups stained for quantification of lipid droplets. Ratio of lipid droplets (Mean ± SD) were significantly lower (p < 0.05) in embryos of VITRO-HIGH compared to VITRO-LOW groups (**F**).

**Figure 6:**
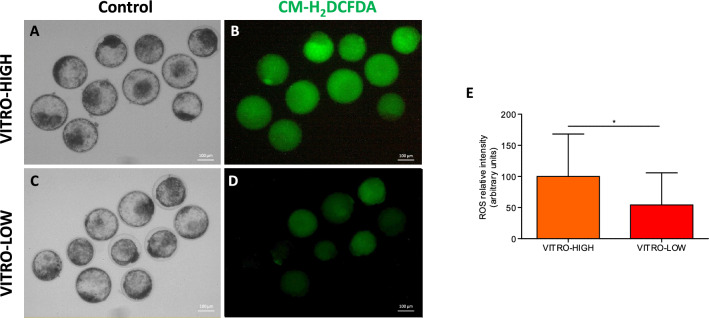
Representative pictures of pools of embryos of VITRO-HIGH (**A**,**B**) and VITRO-LOW (**C**,**D**) groups stained for quantification of ROS. Ratio of ROS (Mean ± SD) was significantly (p < 0.05) higher in embryos of VITRO-HIGH compared to VITRO-LOW groups.

Quantification of lipid droplets revealed no difference between VIVO and VITRO-HIGH embryos (Fig. 5A–C). Conversely, embryos of the VITRO-LOW group demonstrated higher mean values for lipid droplet accumulation (p < 0.05) compared to VITRO-HIGH embryos (Fig. 5D–F). In contrast, analysis of ROS level showed higher relative ROS intensities in embryos of the VITRO-HIGH group compared to embryos of the VITRO-LOW group (p < 0.05), as presented in Fig. 6. All raw data regarding intracellular lipids and reactive oxygen species of this study are included in the supplementary information files (Supplementary data 1).

## Discussion

Average OCR values per embryo measured within our study confirmed those observed in a previous study by Muller and collegues reporting values of 0.85 ± 0.08 pmol/min/embryo for in vitro derived bovine blastocysts^22^. These are close to those obtained in our study with in vitro derived blastocysts showing OCR values of 0.61 ± 0.110 (VITRO-HIGH) or 0.58 ± 0.068 pmol/min/embryo (VITRO-LOW) and VIVO embryos reaching 0.81 ± 0.032 pmol/min/embryo. Considering earlier studies using other measurement strategies to analyse oxygen uptake, the average amount of oxygen uptake per embryo measured within the present study were comparable to these recent studies, too^23–25^. Here, for example Obeidat and colleagues, using electrochemical based sensors, reported values of 1.48 ± 0.3 fmol/s^24^ or Lopes and colleagues,using nanorespirometrie, identified values of 1.3 ± 0.064 fmol/s on single embryo level^23^. In accordance with our results, Thompson et al. also observed differences between in vivo and in vitro derived embryos in terms of OCR^25,26^.

In contrast, however, our study did not obtain contrasting total OCR rates between IVP embryos of different morphokinetics reflecting different embryo qualities. Nonetheless, we suggest that variance in vitality of bovine embryos might be correlated with different total oxygen consumption rates. This dependence, however, might not be detectable between early and late blastocysts, which represent only one criterion reflecting embryo quality among several others. One, explanation might be that total embryo oxygen consumption does not reflect the exact oxygen consumption of mitochondria. Indeed, there are other cell-surface related oxygen consuming processes in cells. Manes and Lai for example identified cell-surface related oxygen consumption by a superoxide generating NAD(P)H oxidase on the surface of rabbit blastocysts^18^. Non-mitochondrial utilization of oxygen in turn has been demonstrated to result in the formation of reactive oxygen species (ROS) and is therefore considered an unfavourable reflector of “bioenergetic health” when present in a high degree^27^. In our experiments, embryos of the VIVO, VITRO-HIGH and VITRO-LOW groups utilized 15.0%, 6.5% and 8.9% of totally consumed oxygen for non-mitochondrial oxygen consumption, respectively, and thus did not differ significantly. This is within the range of a recent study of Muller and colleagues reporting a proportion of roughly 20% for non-mitochondrial respiration in in vitro derived embryos^22^. Also, VIVO, VITRO-HIGH and VITRO-LOW embryos utilized similar average amounts as well as relative proportions of total oxygen uptake for mitochondrial respiration in our study. In summary, the results of our study indicate that differences with respect to embryo viability as a consequence of contrasting environments as well as morphokinetics during early development are not a primarily consequence of differences in terms of total mitochondrial respiration. Although we speculate that sufficient levels of energy generation is of great importance for embryo vitality, it has to be kept in mind that similarity in terms of mitochondrial respiration does not necessarily indicate an equal energy production, as mitochondrial consumed oxygen is only partially coupled with the production of ATP. If statements are to be made about embryonic vitality, it is of interest to characterise the proportions of ATP-linked respiration and its counterpart, the proton leak. Indeed, a strong correlation between morphokinetics as a model for embryo quality of IVP derived embryos and both absolute and relative amounts of ATP-linked respiration was observed within our study. IVP derived embryos of presumably high quality reach the proportion of ATP-linked respiration of ex vivo derived embryos. In contrast, IVP derived embryos of presumably low quality utilized significantly lower proportions of oxygen for ATP-linked respiration compared to IVP embryos of presumably high quality and ex vivo derived embryos. This confirms an earlier study by Noguchi and colleagues reporting contrasting ATP contents between ex vivo and in vitro derived bovine blastocysts^15^. Of impact, our results reveal that fast-developing IVP derived bovine embryos, representing a subpopulation among all IVP embryos being of presumably high vitality, apparently consumed fivefold higher volumes of oxygen for ATP-linked respiration compared to the slowly developing ones representing a subpopulation of presumably low quality. While such a correlation between developmental kinetics and oxygen-utilization for ATP generation has not been reported in the bovine, similar correlations have been reported for murine embryos by Spielmann and colleagues earlier^28^. Accordingly, the coupling efficiency of ATP generation was identified to be much lower in IVP embryos of presumably low quality, utilizing approximately 10% of consumed oxygen, compared to that of IVP embryos of presumably high quality and of ex vivo derived embryos, both utilizing approximately 65% of their consumed oxygen for ATP generation. These results are within the range of earlier studies^22,26,29^. Keeping in mind the low proportion of only 10% of consumed oxygen utilized for ATP-generation in IVP derived embryos of presumably low quality, however, raises the question about the destination of the remaining 90% of consumed oxygen. Among the consumed oxygen not coupled to ATP production, one fraction is known to be required to compensate against proton-leakage which usually exists up to a certain physiological level, as displayed by Brand and colleagues^30^. Surprisingly, absolute and relative volumes of oxygen related to proton leakage did not differ between embryo groups derived from contrasting environments nor by being of different morphokinetics. The values obtained in our study account for 28–32% and are within the range reported recently for in vitro derived bovine embryos^22^. In summary, our results could provide a conceivable explanation for the slower developmental kinetics of blastocysts expanded at day 8.

For a better understanding of embryonic vitality in relation to mitochondrial metabolic activity, it might be also useful to get insight into the maximum mitochondrial potential of embryos allowing them to respond to increased energy demands potentially necessary for further embryonic development, too. In our study, embryo quality indeed correlated with maximal mitochondrial respiration potential. Ex vivo derived bovine embryos reached a significantly higher average maximal mitochondrial respiration compared to slowly developing IVP derived embryos, while there was no difference between ex vivo and fast developing in vitro derived bovine embryos related to high quality. Considering average maximal mitochondrial respiration rates of approximately 2.3, 1.8 and 1.0 pmol per embryo and minute for vivo derived expanded blastocysts, IVP derived embryos of presumably high quality and IVP derived embryos of presumably low quality, respectively, these values were within the range determined earlier for in vitro derived embryos by Mueller and colleagues^22^. In our study, both ex vivo derived as well as fast developing in vitro derived blastocysts showed a significantly higher mitochondrial spare capacity values than slowly developing in vitro derived expanded blastocysts. In other words, bovine embryos of higher quality bear a higher capability to respond to energy demanding conditions for further development compared to those of lower quality. This finding might also explain the observation that higher developmental kinetics of IVP derived bovine embryos go along with higher pregnancy rates after transfer to recipients^31^. These results also potentially explain the high variability in viability among IVP derived bovine embryos, resulting in lower developmental rates both to the blastocysts stage as well as to term after embryo transfer to recipients when compared with their in vivo derived counterparts. Whether this higher mitochondrial spare capacity is due to a higher total number of mitochondria or due to higher extra potential of mitochondria remains unclear in the present study. If lower mitochondrial spare capacities are a consequence of a reduced number of mitochondria being fixed within the unfertilized oocyte this would agree with earlier studies indicating that the mitochondrial copy number of oocytes correlates with their developmental capacity as shown for example by Santos et and colleagues^32^. In fact, there is consensus that a certain threshold of mtDNA copies, indicating the number of mitochondria, is required for sufficient energy production and thus for subsequent embryo development^32–34^. Nonetheless, it should not be overlooked that the ability for further development does not solely depend on the mitochondrial mtDNA content of the oocyte but it is also affected by embryo developmental environment^35,36^. Despite a rather low number of mitochondria at early developmental stages, embryonic development might be possible by up-regulating mitochondrial DNA replication processes at later development stages^34,37^. Thus, embryo quality at blastocyst stage is largely affected by the environmental conditions during early development^2,38^. Taken together, the results of our study revealed that the quality of IVP derived embryos correlates with the maximal mitochondrial respiration potential. IVP derived embryos of presumably high quality closely resembled ex vivo derived embryos in terms of maximal mitochondrial potential whereas IVP derived embryos of presumably low developmental capacity showed a significantly lower potential.

In addition to metabolic outlines, the results of our study demonstrate that IVP embryos of presumably high quality are associated with lower levels of lipid accumulation. Previous studies demonstrated, that higher embryo metabolism rates go along with increased utilisation of endogenous lipid stores via ß-oxidation^39,40^. In agreement, IVP derived embryos of low quality were found to be associated with lower mitochondrial activity and higher lipid accumulation within the present study. This is in line with earlier studies reporting that reduced catabolism of intracellular lipids or even lipid accumulation is a result of insufficient metabolism by mitochondria^41^. Interestingly, we were not able to determine any difference between lipid content of ex vivo derived embryos and IVP derived embryos. This finding is in agreement with those by Annes and colleagues reporting a similar lipid composition in in vivo and in vitro derived embryos, whereas the lipid profile was impacted by different morphokinetics^42^. However, the limited number of analysed in vivo derived embryos in our study has to be considered. Therefore, these findings have to be taken with care since it cannot be fully excluded that differences exist between ex vivo embryos compared to in vitro derived embryos of presumably high quality to some extent. Whether a slower development of IVP derived embryos as a model for a lower embryo quality being associated with higher lipid accumulations and lower mitochondrial metabolism rates are due to lower mitochondrial qualities or due to lower numbers of total mitochondria, however, cannot be ruled out by our results.

Furthermore, the present study obtained significantly lower ROS levels in IVP derived embryos of slower compared to faster developmental kinetics. Conversely, higher levels of ROS have been connected to higher levels of embryo metabolic stress. In that light, the present study demonstrated that higher respiration activity of IVP derived embryos of presumably higher quality go along with lower lipid contents and higher ROS levels. This seems to contradict earlier findings proposing that lower levels of ROS are beneficial for further embryo development and the earlier “quiet embryo hypothesis” by Leese, postulating that a quite embryo metabolism is beneficial^43^. On the other hand, Brand and colleagues pointed out that a specific threshold of ROS is necessary and beneficial for cell signalling and further development^30^. Outside an optimal range, intracellular ROS indeed result in various developmentally regulated modes of embryo demise^44^. ROS level therefore cannot be considered in isolation but must be considered in relation to metabolic activity. Thus, balance between embryo metabolic activity and the level of antioxidant molecules determining ROS level, in other words oxidative homeostasis, might be one of the most important criteria determining quality of an IVP embryo. In light of our results, an intermediate metabolic activity going along with intermediate ROS-level might be most beneficial for further embryo viability as recently introduced by Leese and colleagues by the “goldilocks principal”^45^. Consequently, the results of our study demonstrated that embryo quality of IVP derived embryos as determined by morphokinetics goes along with relatively lower lipid contents and relatively higher ROS level.

Collectively, our study revealed the mitochondrial signature of VIVO embryos, reflecting the “gold-standard” compared to IVP derived embryos of fast and slow morphokinetics reflecting embryos of presumably high and low quality, respectively. The results presented in this work demonstrate that it is usefull to divide embryo mitochondrial oxygen consumption in its features. Although mitochondrial respiration did not differ between groups in the present study, clear differences in terms of energy production and efficiency of it were uncovered. In addition, the present work revealed that in vitro derived embryos of low quality bear an altered mitochondrial potential reflected in a reduced spare capacity. Noteworthy, fast-developing IVP embryos associated with high quality resemble the level of ex vivo derived embryos in important characteristics such as reserve capacity and efficiency of ATP production.

Together, the quality of bovine embryos correlates positively with embryo total oxygen consumption, ATP-linked respiration, efficiency of ATP generation as well as mitochondrial spare capacity. That distinct metabolic fingerprint was found to go along with a relative low lipid content and a relatively high ROS level, supporting the goldilocks principle. Altogether, the present study is the first one reporting typical mitochondrial oxygen consumption signatures related to developmental capacity reflecting contrasting environments of development as well as different embryo qualities based on embryo morphokinetics in bovine embryos. Whether differences in terms of mitochondrial respiration signatures, however, are a cause of contrasting mitochondrial densities per embryo or of contrasting mitochondrial efficiencies should be explored in future studies.

## Material and methods

### Experimental design

In this study, three experimental groups of bovine expanded blastocysts were generated. First, we collected fully in vivo-derived expanded bovine blastocysts (VIVO, n = 32) generated by the artificial insemination of super stimulated Simmental heifers with the semen of a Holstein Friesian bull. The same bull was also used for in vitro fertilization (IVF). These were classified into in vitro derived embryos (IVP) of high quality in case of development to the expanded blastocyst stage by day 7 (VITRO-HIGH, n = 32). Conversely, embryos were classified into IVP embryos of low quality if they reached expanded blastocyst stage at day 8 (VITRO-LOW, n = 48).To analyse potential correlations between bovine embryo metabolic activity and embryo quality, two models for developmental capacity of bovine embryos were used: First, VIVO blastocysts (pools of 8, n = 4 replicates) were compared with VITRO-HIGH (pools of 8, n = 4) by an extracellular FLUX Analyzer (Seahorse XFp, Agilent) to analyse the effect of the environment. Secondly, VITRO-HIGH blastocysts were compared with VITRO-LOW (pools of 8, n = 6). In any case, the extracellular FLUX Analyser was used to analyse the total embryo oxygen consumption rate (OCR) reflecting the extent of oxidative phosphorylation. After measurement of 20 cycles, a mitochondria stress test (XF Cell-MitoStress Test Kit, Agilent) was used to analyse mitochondrial respiration, non-mitochondrial respiration, maximal mitochondrial respiration, proton leakage, mitochondrial spare capacity, ATP-linked respiration as well as efficiency of ATP generation.

### In vivo embryo collection

To generate VIVO blastocysts, Simmental heifers (~ 440 kg) were subjected to a superovulation protocol. Synchronization was performed by administration of 500 mg of cloprostenol, i.m. (Estrumate). This treatment was repeated after 11 days. Two days after each of the cloprostenol treatments, cows received 0.02 mg of GnRH (Receptal). Twelve days after the last GnRH administration, cows received the first of eight consecutive administrations of FSH over the course of 4 days in decreasing dosages (in total, 300–400 mg of FSH equivalent according to body weight; Folltropin, Vetoquinol). At 60 and 72 h after the initial administration of FSH, cows received two treatments of cloprostenol. Finally, 48 h after the first of the two cloprostenol applications, ovulation was induced by 0.02 mg of GnRH and heifers received a first insemination. Artificial insemination was repeated twice within a 12-h interval. Embryos were flushed out of the uterus from heifers that had undergone superovulation treatment 7 days after artificial insemination performed on the day of heat. Briefly, an embryo-flushing catheter (CH15; Wörlein) was fixed in the uterine horns. Subsequently, embryos were flushed out by draining each uterine horn with 500 ml of PBS + 5% NCS. The uterus was flushed using a catheter connected with an embryofilter (Emconfilter 1; no. 04135; Immuno Systems Inc.). Embryos were washed twice in PBS and were evaluated by developmental stage. Only expanded blastocysts were used for further metabolic experiments.

### In vitro embryo production (IVP)

Abattoir derived ovaries were collected and transported to the laboratory in insulated flasks containing 0.9% saline solution (21–30 °C). Upon arrival, cumulus oocyte complexes (COCs) were aspirated from small follicles (2–8 mm) and COCs with a homogenous, evenly granulated ooplasm, with oocytes surrounded by at least three layers of cumulus cells, were transferred to modified Tissue Culture Medium 199 (TCM; Sigma) supplemented with 4.4 mM HEPES, 33.9 mM NaHCO_3_, 2 mM pyruvate, 2.9 mM calcium lactate, 55 µg m^−1^ gentamycin, and 12% (v/v) heat-inactivated ECS. After washing COCs three times, they were cultured in groups of 50 in 400 µl of modified Parker Medium supplemented with 10 µg ml^−1^ FSH (FSH-p; Schering-Plough) at 38.8 °C in a humidified atmosphere with 5% (v/v) CO_2_ in air. Fertilization was performed in Fert-TALP medium^46^ supplemented with 20 µM penicillamine, 10 µl PHE (Hypotaurine-Epinephrin-solution), 6 mg ml^−1^ BSA-*FFA*, 50 µg ml^−1^ gentamycin, and 1 µg ml^−1^ heparin. For IVF, semen of the same sire as used for the generation of VIVO embryos was used. The final concentration of sperm in fertilization droplets was adjusted to 2 × 10^6^ sperm per millilitre. Following 18 h of coculture, the presumptive zygotes, after removal of cumulus cells and sperms, were washed three times and were transferred to in vitro culture. Embryo culture was performed in groups of 50–60 in humidified atmosphere with 5% (v/v) CO_2_ and O_2_ in air at 38.8 °C for up to 8 days in 400 µl of SOFaa medium^47^ supplemented with 6 mg ml^−1^ BSA-*FAF* overlaid with mineraloil, respectively. Only embryos that reached the expanded blastocyst stage by day 7 of culture (VITRO-HIGH) or later at day 8 (VITRO-LOW) were selected for further analysis.

### Determination of embryo basal oxygen consumption rate (OCR)

Oxygen consumption rates were determined by application of an extracellular FLUX Analyzer (Seahorse XFp, Agilent) capturing changes in oxygen concentration via flourometric measurement. Sensors containing Seahorse fluxpaks (Agilent Technology) were incubated overnight at 37 °C in a non-CO_2_ humidified incubator according to the manufacturer’s recommendation. Prior to measurement, the pre-warmed cell plate containing biological material was loaded into the machine according to the manufacturer’s recommendation. All 8 wells of the cartridge were filled with 180 µl of modified SOFaa medium supplemented with 4 mM glucose and 0.4% BSA-FAF but lacking NaHCO_3_ and Phenolred (SOF Seahorse). Among these, two plate specific ‘blank’ cell-free wells containing solely medium were used to account for environmental changes and flux of oxygen in the absence of cells, whereas 6 wells were used for the analysis of pools of 8 expanded blastocysts. To place expanded blastocysts in the correct position directly under the fluorophore sensor, embryos were caged through a custom prepared embryo positioning system (Suppl. Fig. 2). Briefly, the system consists of a custom prepared polyester mesh (150 µm mesh size, Labomedic) to avoid disaggregation of the embryos from the bottom of the well. The mesh was previously punched out by a biopsy punch (Ø 4 mm, mediware) and washed three times in SOFSeahorse. To further avoid movement of embryos from the centre of the well to the periphery, a specific insert providing a free centre area for the embryos was used (Suppl. Fig. 2). After the calibration according to the manufacturer’s recommendation and equilibration (15 min), baseline measurement of embryo group OCR was determined using a protocol alternating between a 3-min measurement period and a 30 s re-equilibration period (20 cycles). The measurement period involves the lowering of a sensor, creating a microchamber, for measurement. This is followed by a 30 s period in which the probe is lifted, and the 180 μl well re-equilibrates. The mean value of OCR per well (20 cycles) was noted and normalised to embryo per well as total embryo oxygen consumption given in pmol/min/embryo.

### Measurement of mitochondrial oxygen consumption signature:

To determine mitochondrial respiratory characteristics, mitochondrial inhibitors (Mito-Stress Test Kit, Agilent) were loaded immediately before calibration. Briefly, mitochondrial inhibitors (Oligomycin, Carbonyl cyanide-p-trifluoromethoxyphenylhydrazone (FCCP) and Rotenone/Antimycin A) were dissolved in SOFSeahorse according to manufacturer’s guidelines (XF Cell-Mito Stress Test Kit, Agilent). Inhibitors were diluted in warmed analysis media (SOF Seahorse) and loaded into the cell plate (injection ports A–C) within 30min of starting the assay. Concentrations and volumes for mitochondrial inhibitorswere1.5 µM and 20 µl for Oligomycin (injection port A), 4.0 µM and 22 µl for FCCP (injection port B) and 2.5 µm and 25 µl for Rotenone/Antimycin A (injection port C).

Serial injection of (1) oligomycin, (2) FCCP, and (3) a combination of antimycin A and rotenone each separated by 5 measurement cycles was used for mitochondrial parameters (Supplementary Fig. 1). In detail, the difference between total embryo oxygen consumption and OCR after injection of oligomycin indicates ATP-linked respiration given in pmol/min/embryo. The OCR after injection of FCCP indicates the average maximal mitochondrial respiration per embryo (pmol/min/embryo). The OCR after injection of antimycin A and rotenone indicates the average non-mitochondrial respiration per embryo (pmol/min/embryo). Similarly, the difference in OCR between average total embryo oxygen consumption and average non-mitochondrial respiration per embryo indicates the average mitochondrial respiration per embryo (pmol/min/embryo). Finally, the difference between OCR values after injection of FCCP and after injection of antimycin A and rotenone indicates the average proton leakage per embryo (pmol/min/embryo). The proportion of mitochondrial respiration relative to total embryo oxygen consumption determines mitochondrial respiration (%). Similarly, the proportion of ATP-linked respiration relative to mitochondrial respiration determines the efficiency of ATP generation; and the proportion of maximal mitochondrial respiration relative to mitochondrial respiration determines mitochondrial spare capacity (%). In any case, OCR values (pmol/min) determined by the software of the seahorse XFp within the modified measurement wells (embryo cage) containing a pool of 8 embryos were corrected for volume with the factor 6.425 and divided by 8 to get the average OCR on embryobasis (pmol/min/embryo).

### Quantification of embryonic reactive oxygen species (ROS)

Quantification of ROS content was done by using DCFDA (Sigma D-6883). Groups of expanded blastocysts of VITRO-HIGH and expanded blastocysts of VITRO-LOW, were incubated for 30 min in PBS containing 0.1% PVA and 5 mM DCFDA, in the dark at 37 °C. To avoid size-dependent accumulation of the signal in the blastocoelic cavity only expanded blastocysts were analyzed and great care was taken to ensure that variability in terms of blastocyst diameter were homogeneous within and across groups. After three rounds of washing in PBS containing 0.1% PVA, brightfield and fluorescence pictures were taken by an inverted microscope (DM IRBLeica) and a digital camera (AxioCam ERc5s, Zeiss) of pools of ten expanded blastocysts with the help of a special software (ZenCore2 blue edition, Zeiss). Software settings were identical for subsequent stainings (objective × 5, Time to take photo: 2000 ms; Exposure 200 s; Contrast: Black: 0, Gamma: 1 and White: 255). The fluorescence intensities of embryos stained for ROS were analysed by ImageJ software (version 1.47; National Institutes of Health, Bethesda, MD, USA) and normalized to expanded blastocyst of VITRO-HIGH.

### Quantification of embryonic lipid droplet content

To determine the lipid content of expanded blastocysts derived from different experimental groups, embryos were stained with Oil Red and were evaluated under a fluorescence microscope as described for cell cultures elsewhere. Briefly, a 0.5% Oil Red (ORO; Sigma no. O0625) solution in isopropanol (100%) was prepared as stock solution. For every use, Oil Red stock solution was diluted in distilled water (60%:40%) to prepare an ORO working solution. For Oil Red staining, blastocysts were washed three times in PBS containing 1% polyvinyl alcohol, followed by fixation in paraformaldehyde (4%) for 2 min. Thereafter, fixed blastocysts were transferred to Oil Red working solution for 1 h at 25 °C. Following that, expanded blastocysts were washed three times in PBS containing 0.1% polyvinyl alcohol prior to taking photos with a phase-contrast microscope. Photos were taken with AxioCam ERc5 and Software ZenCore (Zeisse), under phase contrast (PH) with objective × 10 (mircoscop DM IRB, Leica). The red area content related to the diameter of embryos stained for lipid droplets were analysed by ImageJ software (version 1.47; National Institutes of Health, Bethesda, MD, USA) and normalized to those of the VITRO-HIGH group.

### Statistical analysis

For data analysis, the Wave Software (Agilent) was used to convert data in an excel file followed by data analysis in R. Cell-MitoStress Test mitochondrial parameters were determined according to manufacture guidance (Agilent). The following mitochondrial parameters were determined: basal embryo oxygen consumption can be separated in non-mitochondrial respiration and mitochondrial respiration. Moreover, determination of maximal respiration, proton leakage, ATP-linked respiration was possible and enables further calculation of relative mitochondrial spare capacity and ATP coupling efficiency. Results are reported as average OCR absolute values per embryo (raw data) given in pmol/min/embryo over time, standardised to VITRO-HIGH group (ratio) or reported as proportions relative to VITRO-HIGH group (%). To analyse differences between contrasting embryo groups in terms of different OCR values, analysis of variance(ANOVA) with bonferroni post hoc test was used. P-values p < 0.05 were considered significant. Pictures for quantification of ROS were analyzed according to grey values and pictures for quantification of lipid droplets (Oil-Red stain) were analysed in relation to red area content by ImageJ software (version 1.47; National Institutes of Health, Bethesda, MD, USA). In both cases, graphical estimation of normal distribution and the F-test for examination of variance homogeneity were used, followed by an unpaired t-test for pairewise comparison. Graphics are prepared with Graph-Pad Prism (Dotmatics).

### Ethics declarations

The present study is reported in accordance with ARRIVE guidelines. All methods were performed in accordance with the relevant guidelines and regulations. Animal handling for collection of in vivo derived embryos was carried out in accordance with the German Law of Animal Protection (TierSchG&TierSchVersV). All experimental protocols performed on cows in this study were approved by the state office for Nature, Environment, and Consumer Protection of North Rhine-Westphalia, Germany (Landesamt für Natur, Umwelt und Verbraucherschutz Nordrhein-Westfalen, Deutschland) under license number 84–02.04.2014.A499.

### Supplementary Information

Supplementary Figure 1.

Supplementary Figure 2.

Supplementary Information.

## Data Availability

All data generated or analysed during this study are included in this published article and its supplementary information files.
