# Supplementary material for: The mitochondrial respiration signature of the bovine blastocyst reflects both environmental conditions of development as well as embryo quality
Source: Sci Rep. 2023 Nov 8;13:19408. doi: 10.1038/s41598-023-45691-2 (PMC10632430; doi:10.1038/s41598-023-45691-2)
Supplement: Supplementary file 1 — Supplementary Figure 1. [file 41598_2023_45691_MOESM1_ESM.pdf]

Kurzella et al., Suppl. Fig. 1

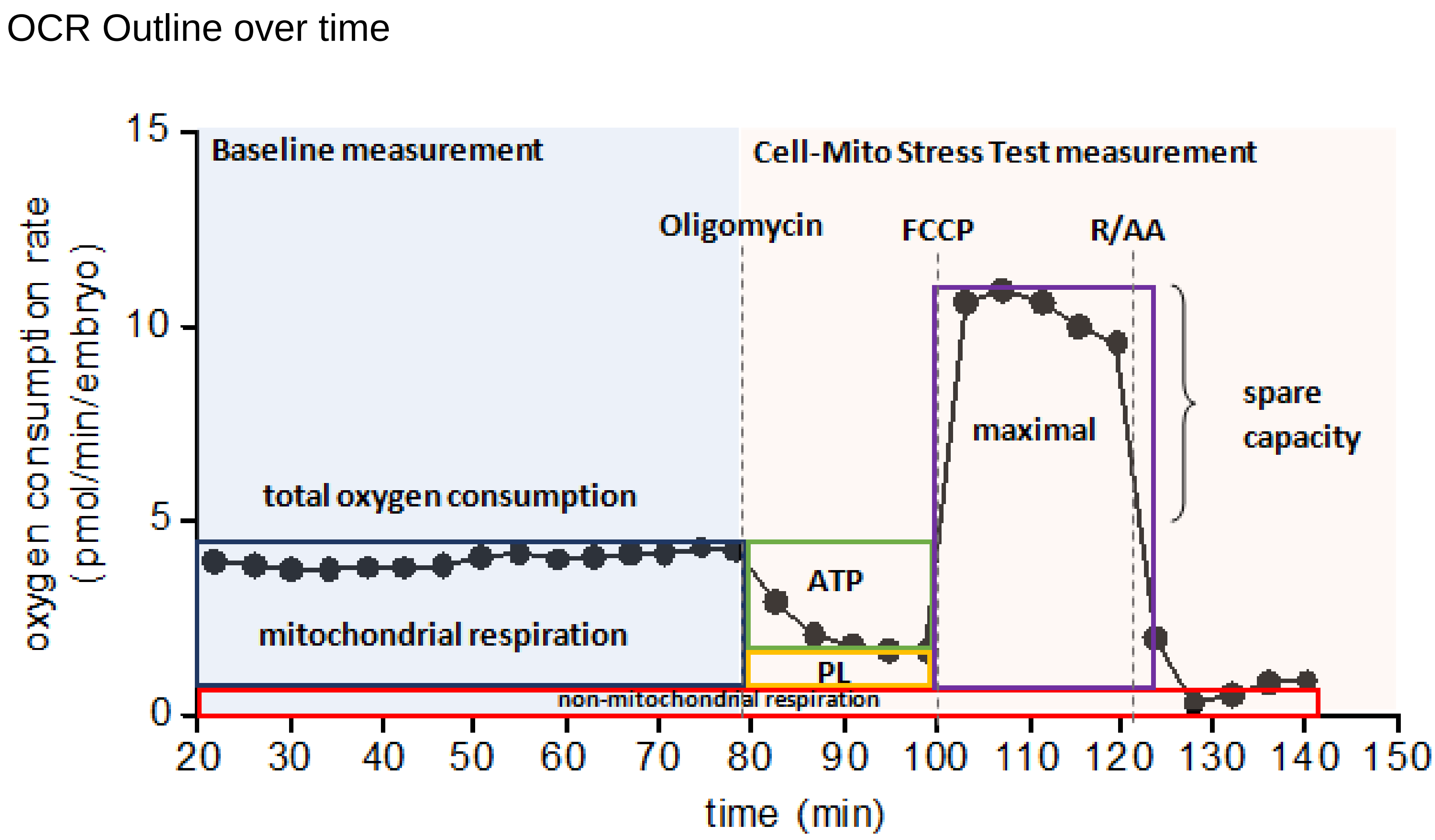

Graphical illustration of oxygen consumption rate during baseline measurement and Cell-Mito Stress Test: Graphic demonstrates Cell-Mito Stress Test Parameter: total oxygen consumption; mitochondrial respiration; non-mitochondrial respiration; Proton Leakage (PL) and ATP-linked respiration (ATP); maximal respiration and Spare Capacity.
