# Supplementary material for: The mitochondrial respiration signature of the bovine blastocyst reflects both environmental conditions of development as well as embryo quality
Source: Sci Rep. 2023 Nov 8;13:19408. doi: 10.1038/s41598-023-45691-2 (PMC10632430; doi:10.1038/s41598-023-45691-2)
Supplement: Supplementary file 2 — Supplementary Figure 2. [file 41598_2023_45691_MOESM2_ESM.pdf]

Kurzella et al., Suppl. Figure 2

The embryo cage

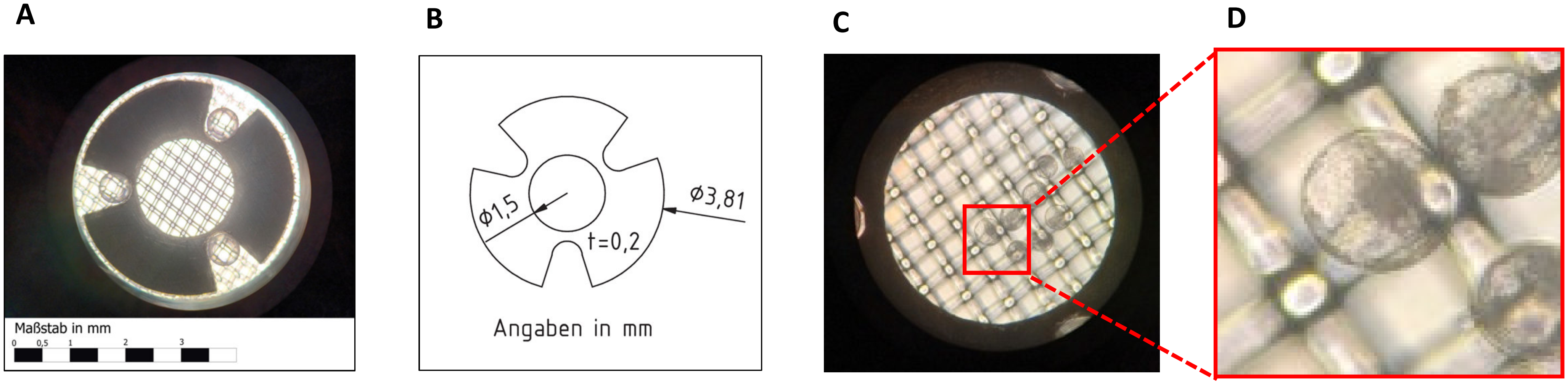

Photography of the embryo cage; consisting of horizontal embryo confinement by a grid (mesh size: 150 µm) and vertical embryo confinement created by a metal insert enabling stable positioning of embryo directly under the sensor necessary for reliable and precise measurement (A). Technical drawing of the metal insert (B). A group of expanded blastocysts positioned by embryo cage (C). Detailed view on expanded blastocysts (D).
